# Temporal biomarker profiles and their association with ICU acquired delirium: a cohort study

**DOI:** 10.1186/s13054-018-2054-5

**Published:** 2018-05-25

**Authors:** Koen S. Simons, Mark van den Boogaard, Eva Hendriksen, Jelle Gerretsen, Johannes G. van der Hoeven, Peter Pickkers, Cornelis P. C. de Jager

**Affiliations:** 1 Department of Intensive Care Medicine, Radboud University Medical Center, Geert Grooteplein 21, 6500 HB Nijmegen, The Netherlands; 2 Department of Intensive Care Medicine, Jeroen Bosch Hospital, Henri Dunantstraat 1, 5200 ME ‘s-Hertogenbosch, The Netherlands

**Keywords:** Delirium, ICU, Critical illness, Biomarkers, Serial measurement

## Abstract

**Background:**

Neuroinflammation is thought to play an important role in the pathogenesis of ICU-acquired delirium, but the association between inflammatory and brain-specific proteins and ICU delirium is poor. We investigated whether or not serial determinations of markers may improve this association.

**Methods:**

Critically ill patients with a high risk of ICU delirium and with an ICU length of stay of at least 6 days were included in the study. Blood was drawn on days 1, 2, 4 and 6 after ICU admission and analyzed for different markers of inflammation and several brain proteins. Differences in courses over time prior to and following the onset of delirium and absolute differences over time were analyzed in patients with and without delirium using repeated measurement analysis of variance. In addition, a cross-sectional analysis of levels of these markers before the first onset of delirium was performed.

**Results:**

Fifty patients were included in this study. In the longitudinal analysis, there were no differences in the levels of any of the markers immediately prior to and following the onset of delirium, but overall, median levels of adiponectin (9019 (IQR 5776–15,442) vs. 6148 (IQR 4447–8742) ng/ml, *p* = 0.05) were significantly higher in patients with delirium compared to patients without delirium. In the cross-sectional analysis, median levels of the brain protein Tau (90 (IQR 46–224) vs. 31 (IQR 31–52) pg/ml, *p* = 0.009) and the ratio Tau/amyloid β_1–42_ (1.42 ((IQR 0.9–2.57) vs. 0.68 (IQR 0.54–0.96), *p* = 0.003) were significantly higher in patients with hypoactive delirium compared to patients without. Levels of neopterin (111 (IQR 37–111) vs. 29 (IQR 16–64) mmol/l, *p* = 0.004) and IL-10 (28 (IQR 12–39) vs. 9 (IQR 4–12) pg/ml, *p* = 0.001) were significantly higher in patients with hypoactive delirium compared to patients with mixed-type delirium.

**Conclusions:**

While there are differences in markers (adiponectin and several brain proteins) between patients with and without delirium, the development of delirium is not preceded by a change in the biomarker profile of inflammatory markers or brain proteins. Patients with hypoactive delirium account for the observed differences in biomarkers.

**Trial registration:**

ClinicalTrials.gov, NCT 01274819. Registered on 12 January 2011.

**Electronic supplementary material:**

The online version of this article (10.1186/s13054-018-2054-5) contains supplementary material, which is available to authorized users.

## Background

ICU-acquired delirium is a frequently occurring problem in critically ill patients and is independently associated with a myriad of negative short-term and long-term outcomes [1–3]. While recent research shows that many risk factors are associated with the development of delirium, its pathogenesis is still incompletely understood and involves a combination of predisposing and precipitating factors [4]. In recent years, the inflammatory response as a trigger for brain damage and its clinical substrate, delirium, has been a subject of investigation. This neuro-inflammatory hypothesis assumes a systemic inflammatory response, which stimulates release of cytokines in the brain by microglial cells. The cerebral cytokine release leads to a spectrum of clinical symptoms, ranging from a relatively mild sickness behavior syndrome to full-blown delirium [5, 6]. Indeed, associations between levels of pro-inflammatory markers and the presence of delirium have been found in ICU and non-ICU patients [7–10]. Also serum levels of other biomarkers involved in the inflammatory response, such as neopterin and monocyte chemoattractant protein-1 (MCP-1) are found in higher concentrations in patients with delirium [11, 12]. Interestingly, markers of brain damage have also been associated with delirium [9, 13, 14].

Levels of pro-inflammatory and anti-inflammatory cytokines and adiponectin are also associated with delirium in ICU patients [9, 10]. Since the levels of biomarkers and the development of delirium may change over time, serial measurements of biomarkers may further elucidate the interplay between inflammation and neuronal injury. However, up to now, only a limited number of studies with a time-series design have been performed in ICU patients. There appear to be higher levels of IL-6 in patients with delirium and an association between S-100B concentrations and duration of delirium [15, 16]. However, only a limited number of biomarkers or a limited number of time points have been analyzed. To gain more insight into the prediction and development of delirium per se, the aim of our study therefore was to determine whether levels of inflammatory biomarkers, proteins, and brain proteins, follow a different course in patients with delirium compared to controls, before and after the actual development of delirium. Additionally, a cross-sectional analysis of these markers prior to the first occurrence of delirium was performed, thereby discriminating between the subtypes of delirium.

## Methods

### Study design

This biomarker study was part of the “Dynamic Light Application to reduce ICU-acquired delirium” (DLA) study, ClinicalTrials.gov number NCT 01274819), which was a single-center randomized controlled trial, published elsewhere [17]. In this study no difference in any measured outcome was found between the intervention and the control group. The study was approved by the regional medical ethical committee (registration number M392 NL 34780.028.10, Medisch Ethische Toetsing Onderzoek bij Patiënten en Proefpersonen (METOPP), Tilburg, the Netherlands). A preplanned longitudinal case-control subgroup analysis of biomarkers in patients with a high risk of developing delirium in the ICU was part of the original protocol.

### Patients and procedures

Inclusion and exclusion criteria for the DLA study have been reported previously [17]. Between 2011 and 2013 a total of 734 patients, who were not suffering from delirium at the time of inclusion, were included in the DLA study. For our biomarker sub-study we planned to evaluate approximately 200 patients with a high risk of delirium. Patients were therefore eligible if they had predicted risk of delirium in the ICU > 40% using the validated PRE-DELIRIC model [18]. Since we wished to investigate if there was a biomarker that showed an increase just before the development of delirium, only patients with a length of stay of at least 6 days were included and patients that remained comatose during the first 7 ICU days were excluded. For the same reason patients who developed delirium after 6 days of ICU admission were also excluded. Blood was drawn from an indwelling arterial catheter on the morning after their ICU admission at 0800 h (admission day = 1), and, at the same time, on days 2, 4 and 6. Blood samples were centrifuged and plasma was stored at − 70 °C until analysis.

### Delirium assessment

Delirium was assessed using the Confusion Assessment Method for the ICU (CAM-ICU). This widely used and validated instrument, which has good performance [19], was performed at least three times per day as part of normal care. During the study period, the quality of the CAM-ICU assessments was tested bimonthly by expert delirium nurses to check for inter-observer reliability, which remained good during the study, with a mean Cohen’s κ of 0.79 (*n* = 178). Patients were considered as having delirium if the CAM-ICU was positive at least once within the first 7 days of their ICU admission. Delirium was assessed on a daily basis; if at least one CAM-ICU screening was positive on any day, this was considered to be a “delirium day”. It was then determined if there was a peak in biomarkers just before the onset of delirium. Importantly, in the setting of sedative use, patients with only one positive CAM-ICU screening during their admission, with a Richmond Agitation and Sedation Scale (RASS) score of − 1/− 3, were considered to have rapidly reversible sedation-related delirium [20]. These patients were not considered as truly delirious patients. Patients who developed delirium after 6 days in the ICU and patients who were comatose during the measurement period (defined as a persistent RASS score of − 4 or less) were excluded. In all patients the subtype of delirium was defined by means of the RASS score according to the Peterson criteria [21]; if the RASS was persistently zero or less during the time that delirium was present, this was considered to be hypoactive delirium, if not directly related to the use of sedatives. If the RASS was below and above zero during delirium, this was considered to be mixed-type delirium, and patients with delirium who consistently had a RASS above zero were identified as having hyperactive delirium.

### Biomarkers

Concentrations of the pro-inflammatory cytokines tumor necrosis factor (TNF)-α, interleukin (IL)-6 and IL-1β, anti-inflammatory cytokine IL-10 and the chemotactic cytokine MCP-1 were determined in ethylenediaminetetraacetic (EDTA)-anticoagulated plasma by a simultaneous multiplex immunoassay, according to the manufacturer’s instructions (Bio-Plex, BioRad, Hercules, CA, USA). The lower detection limit for all cytokines was 4 pg/mL. Concentrations of adiponectin, neopterin and total tau-protein were determined in edta-anticoagulated plasma using enzyme-linked immunosorbent assay (ELISA), according to the manufacturer’s instructions (R&D systems, Abingdon, UK, IBL international GmbH, Hamburg, Germany and Life technologies, Bleiswijk, The Netherlands). The lower limits of detection were 390 ng/mL for adiponectin, 1.35 nmol/L for neopterin and 31.25 pg/mL for Tau-protein. Levels of adiponectin were corrected for the patient’s body mass index. The brain-specific proteins full-length amyloid β_1–42_ and _1–40_ (Aβ_1–42_ and Aβ_1–40_) were determined in EDTA plasma by using a simultaneous Luminex assay (INNO-BIA plasma Aβ forms; Fujirebio, Gent, Belgium). The lower limit of detection was 18 pg/mL for both Aβ_1–40_ and Aβ _1–42_.

### Statistics

The course over time in levels of the markers was evaluated prior to or following delirium occurrence. The day of delirium occurrence was taken as a reference point, and the days that blood was drawn (admission days 1, 2, 4 and 6) were recoded based on this time point. For example, if delirium occurred on admission day 4 biomarkers were analyzed on time point t-3 (admission day 1), t-2 (admission day 2), t (admission day 4) and t + 2 (admission day 6). For comparison with the control group, the median day of delirium occurrence in the delirium group (i.e. day 3) was used for the non-delirious patients. This means that in these patients blood was drawn on t-2 (admission day 1), t-1 (admission day 2), t + 1 (admission day 4) and t + 3 (admission day 6) (see Additional file 1).

A cross-sectional analysis of differences in levels of biomarkers one day before occurrence of delirium was performed. Average values of the biomarkers before the first occurrence of delirium were calculated by taking the exact value of the biomarker if it was taken on the day before the first occurrence of delirium or by averaging the value of the biomarker taken the day before and on the day of the occurrence of delirium in case delirium occurred on days that no blood was drawn for analysis (i.e. day 3). For patients, that did not develop delirium, the value of the biomarker on the day before delirium occurred, on average in the delirium group, was used. In cases where delirium was already present on the day of the first time point, the value of that sample was used (Additional file 1).

Differences in baseline characteristics between delirium and non-delirium patients were tested using the chi-square (χ^2^) test for categorical variables and Student’s *t* test or the Mann-Whitney U test for normally or non-normally distributed variables, respectively. The relationship between the biomarker levels over time and the presence of delirium was assessed using repeated measures analysis of variance (RM-ANOVA) whereby the between-subject factor group delirium and non-delirium was reported, and which was adjusted for variables that were significantly different between patients with and without delirium. Due to the fact that the assumptions of homogeneity and sphericity were violated, the Greenhouse-Geisser correction was used. Since several biomarkers had a skewed distribution, the biomarker data were log-transformed allowing us to perform RM-ANOVA. Due to the exploratory nature of the longitudinal part of this study no correction for multiple testing was performed in order to increase the sensitivity to detect differences between groups. Differences between non-delirium and the subtypes of delirium and the biomarkers were tested using the Mann-Whitney U test. All statistical analyses were performed using SPSS version 22.

## Results

**Fig. 1:**
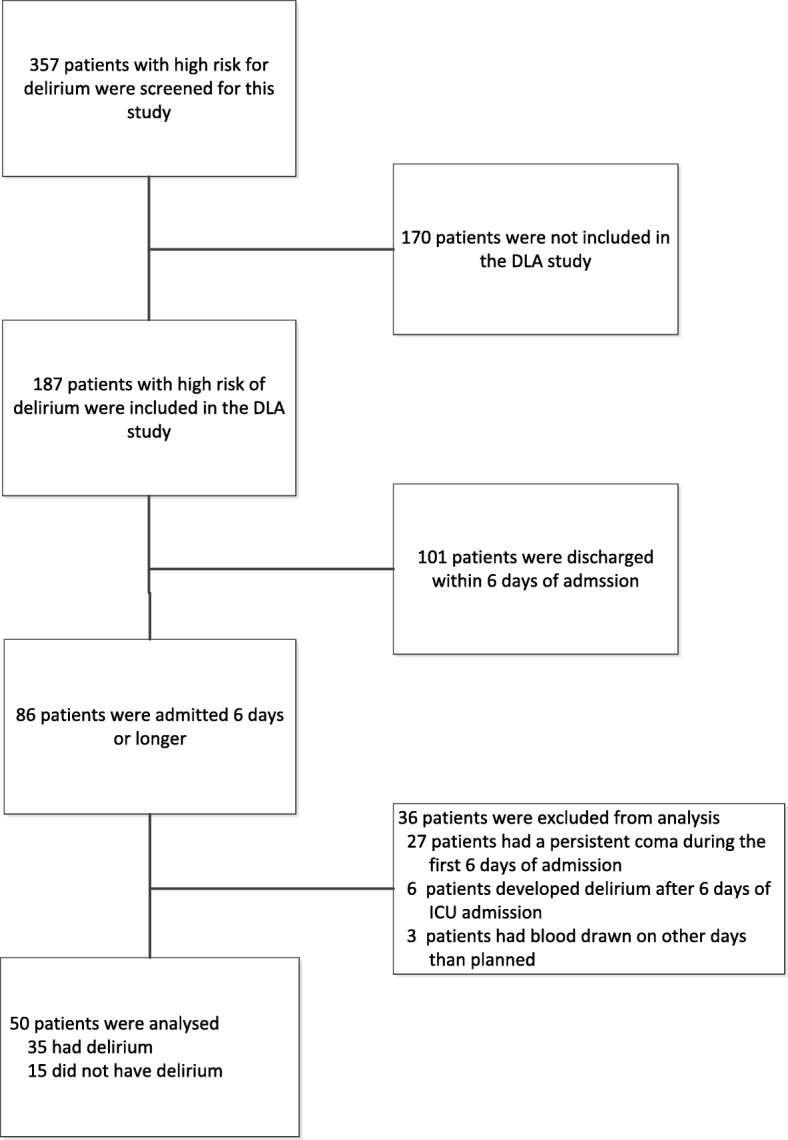
Enrollment scheme: selection of patients for the current study; to enable serial sampling, only patients with an ICU length of stay of at least 6 days were selected. DLA, dynamic light application

Between July 2011 and September 2012, 187 patients included in the DLA study who had a high risk of delirium were evaluated for this study. Of these patients, 101 were discharged within 6 days of ICU admission and therefore a total of 86 patients were included in this study (see Fig. 1). Of these patients, 27 (31%) were in a persistent coma during the first week, 6 patients (7.0%) developed delirium after 6 days of ICU admission and in 3 patients (3.5%) blood was drawn on days other than those scheduled. As a result, 50 patients were analyzed for this longitudinal study, with a mean age of 72 years (± 10.3) of whom 36 patients (74%) were male (Table 1). Delirium occurred in 35 patients (70%). Patients in the delirium group were significantly older than non-delirious patients (73 vs. 67 years, *p* = 0.03) but there were no significant differences between the two groups in baseline demographic characteristics, although more patients tended to be admitted with sepsis in the delirium group compared with the non-delirium group (46% vs. 20%, *p* = 0.08).

**Table 1:** Baseline characteristics of the study population

	Delirium (*n* = 35)	No delirium (*n* = 15)	Differences (*p* value)
Age in years, mean (SD)	73 (9.9)	67 (10)	0.03
Male, *n* (%)	23 (66)	13 (87)	0.12
Admission type:			
Surgical (%)	8 (23)	3 (20)	
Medical (%)	26 (74)	10 (67)	0.35
Neurotrauma (%)	1 (3)	2 (13)	
APACHE-II score (SD)	26 (7)	28 (9)	0.42
Sepsis (patients), *n* (%)	16 (46)	3 (20)	0.08
PRE-DELIRIC score (mean, SD)	78 (18)	74 (19)	0.45
History of cognitive disorder, *n* (%)	4 (11)	1 (7)	0.52
Diabetes mellitus, *n* (%)	5 (14)	1 (7)	0.41
COPD, *n* (%)	1 (3)	2 (13)	0.21
Acute kidney injury, *n* (%)	12 (34)	3 (20)	0.25
Cardiovascular disease, *n* (%)	1 (3)	1 (7)	0.51
Use of steroids, *n* (%)	18 (51)	8 (53)	0.57
Study arm (DLA), *n* (%)	19 (54)	8 (53)	0.6
Intubation, *n* (%)	31(89)	14(93)	0.52
Hours of mechanical ventilation, median (IQR)	158 (96–281)	194 (116–383)	0.58
LOS-ICU in days, median (IQR)	12 (10–19)	15 (9–24)	0.45
LOS-hospital in days, median (IQR)	30 (17–42)	35 (19–50)	0.79
Hospital mortality, *n* (%)	12 (34)	3 (20)	0.25

Hypoactive delirium was present in 11 patients (31%), mixed-type delirium in 23 patients (66%) and hyperactive delirium in 1 patient (3%). There were no significant differences in ICU or hospital length of stay or in hospital mortality between patients with and without delirium (Table 1).

### Longitudinal analysis

**Fig. 2:**
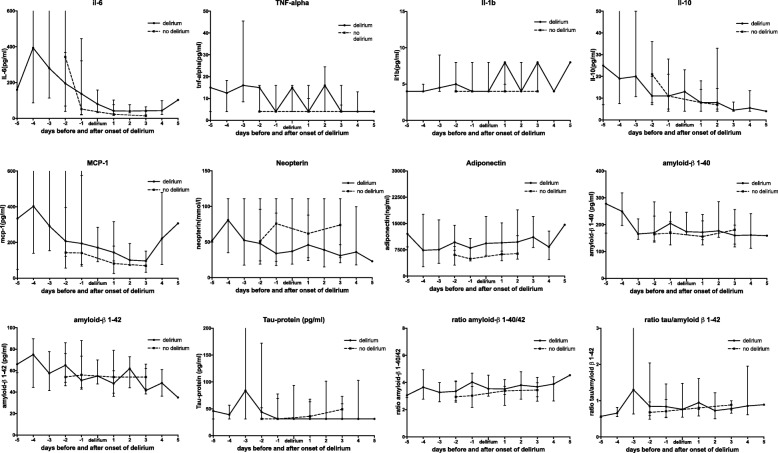
Time course of median values of biomarkers before and after the occurrence of delirium. 0 denotes the day of first the occurrence of delirium

The median day of development of delirium was day 3 (IQR 2–4) after ICU admission. There were no differences in the levels of the markers over the course over time prior to the onset of delirium, or in the time course following the onset of delirium between patients who did or did not develop delirium (Table 2 and Fig. 2).

**Table 2:** Median levels (interquartile range) of biomarkers over time; *p*-values indicate the between-subject differences between delirium and non-delirium groups

	Delirium (*n* = 35)				Non-delirium (*n* = 15)				
	Day 1	Day 2	Day 4	Day 6	Day 1	Day 2	Day 4	Day 6	*p* value
Inflammation									
TNF a (pg/mL)	10 (4–16)	4 (4–16)	4.0 (4–16)	4.0 (4–16)	4.0 (4–16)	4.0 (4–16)	4.0 (4–16)	4.0 (4–16)	0.24
IL-6 (pg/mL)	250 (82–1300)	163 (51–365)	75 (33–130)	43 (26–77)	343 (39–1171)	52 (20–446)	23 (16–102)	14 (11–39)	0.29
IL-1 beta (pg/mL)	4 (4–8)	4 (4–8)	4 (4–8)	4 (4–8)	4 (4–8)	4 (4–4)	4 (4–5)	4 (4–4)	0.13
IL-10 (pg/mL)	12 (7–25)	12 (4–24)	9 (5–18)	7 (4–14)	21 (8–36)	11 (5–21)	8 (4–18)	7 (5–33)	0.82
Neopterin (mmol/L)	37 (18–91)	50 (19–99)	45 (21–111)	39 (22–101)	51 (19–111)	76 (23–111)	62 (28–111)	74 (28–111)	0.18
MCP-1 (pg/mL)	233 (141–642)	239 (97–630)	171 (101–369)	120 (74–263)	143 (56–645)	141 (67–575)	82 (26–180)	70 (33–95)	0.08
Adiponectin (ng/mL)	7821 (4421–13,910)	8986 (4775–12,880)	9737 (6169–15,416)	10,379 (6486–20,695)	6065 (3163–8466)	4927 (4345–8023)	6232 (4393–9457)	6404 (4880–11,550)	0.05
Brain									
Aβ_1–42_ (pg/mL)	58 (46–77)	56 (42–79)	53 (41–64)	55 (38–68)	54 (46–86)	56 (44–88)	54 (39–79)	54 (43–62)	0.74
Aβ_1–40_ (pg/mL)	179 (164–247)	181 (155–280)	177 (146–243)	177 (146–246)	165 (135–232)	169 (125–212)	156 (125–238)	181 (128–257)	0.61
Ratio Aβ_1–40/42_	0.28 (0.24–0.36)	0.28 (0.23–0.32)	0.28 (0.23–0.35)	0.26 (0.22–0.36)	0.34 (0.25–0.38)	0.33 (0.27–0.46)	0.30 (0.27–0.43)	0.29 (0.25–0.34)	0.37
Tau-protein (pg/mL)	31.25 (31.25–59)	31.25 (31.25–120)	31.25 (31.25–101)	31.25 (31.25–87)	31.25 (31.25–52)	31.25 (31.25–69)	36 (31.25–63)	49 (31.25–73)	0.19
Ratio Tau/ Aβ_1–42_	0.68 (0.50–1.19)	0.83 (0.56–1.43)	0.90 (0.59–1.70)	0.89 (0.74–1.29)	0.68 (0.59–0.96)	0.71 (0.53–1.03)	0.80 (0.58–1.42)	0.88 (0.65–1.36)	0.20

Over time, levels of adiponectin remained significantly higher in patients with delirium compared to those without delirium (Table 2 and Additional file 2). Within-subject analysis showed that levels of IL-6, IL-10 and MCP-1 decreased significantly over time in both groups, but were not different between patients with and without delirium. There were no significant differences between groups in any of the other markers over time. Values of Tau-protein, Aβ_1–40_ and Aβ_1–42_ remained stable during the measurement period: 178/200 (89%) of the measurements of IL-1β, 126/200 (63%) of the measurements of TNF-α and 48/200 (24%) of the measurements of neopterin were below the detection limit.

### Cross-sectional analysis

#### Patients with versus patients without delirium

In three patients, delirium occurred before the first blood sampling and in these cases the first value of the biomarkers was used for analysis. Since only one patient was diagnosed with a hyperactive form of delirium, we did not include this patient in the subgroup analysis. We found no differences in levels of inflammatory or brain-specific markers one day before the development of delirium (see Table 3).

**Table 3:** Cross-sectional analysis of biomarkers one day prior to clinical occurrence of delirium; data are expressed as median (IQR)

	No delirium (*n* = 15)	Delirium (*n* = 35)	Hypoactive delirium (*n* = 11)	Mixed delirium (*n* = 23)	Hyperactive delirium (*n* = 1)
Inflammation					
TNF a (pg/mL)	4 (4–16)	4 (4–16)	4 (4–16)	4 (4–16)	4
IL-6 (pg/mL)	343 (39–1171)	212 (75–412)	234 (195–461)	109 (51–365)	981
IL-1 beta (pg/mL)	4 (4–6)	4 (4–8)	4 (4–8)	4 (4–8)	8
IL-10 (pg/mL)	19 (8–28)	12 (5–28)	28 (12–39)^a^	9 (4–12)	20
Neopterin (mmol/L)	51 (19–111)	36 (23–95)	111 (37–111)^b^	29 (16–64)	24
MCP-1 (pg/mL)	143 (56–620)	239 (117–547)	454 (141–596)	168 (90–350)	953
Adinopectin (ng/mL)	6065 (3163–8466)	7997 (4421–12,880)	7391 (3952–10,499)	7997 (5014–16,099)	8212
Brain					
Aβ_1–42_ (pg/mL)	54 (46–86)	57 (46–70)	63 (45–81)	55 (46–67)	70
Aβ_1–40_ (pg/mL)	174 (135–227)	200 (152–272)	229 (200–274)	167 (142–247)	194
Ratio Aβ_1–40/42_	0.34 (0.25–0.4)	0.26 (0.23–0.35)	0.26 (0.19–0.31)	0.27 (0.23–0.38)	0.36
Tau-protein (pg/mL)	31.25 (31.25–52)	31.25 (31.25–101)	90 (46–224)^c^	31.25 (31.25–75)	31.25
Ratio tau/Aβ_1–42_	0.68 (0.54–0.96)	0.91 (0.51–1.48)	1.42 (0.9–2.57)^d^	0.66 (0.5–1.36)	0.45

^a^*p* = 0.001 for the difference between hypoactive delirium and mixed-type delirium

^b^*p* = 0.004 for the difference between hypoactive delirium and mixed-type delirium

^c^*p* = 0.009 for the difference between hypoactive delirium and no delirium

^d^*p* = 0.003 for the difference between hypoactive delirium and no delirium

#### Differences between patients with subtypes of delirium and patients without delirium

When differentiating between clinical subtypes of delirium, levels of Tau-protein and the ratio of Tau/Aβ_1–42_ was significantly higher in the hypoactive delirium group compared to the non-delirium group (median 90 (IQR 46–224) vs. 31.25 (IQR 31.25–52) pg/mL, *p* = 0.009 and median 1.42 (IQR 0.90–2.57) vs. 0.69 (IQR 0.54–0.96), *p* = 0.003, respectively; see Table 3). Additionally, in the subgroup of patients with hypoactive delirium, levels of neopterin and IL-10 were significantly higher than in the mixed-type delirium group (median 111 (IQR 37–111) vs. 29 (IQR 16–64) mg/L, *p* = 0.004 and median 28 (IQR 12–39) vs. 9 (IQR 4–12) pg/mL, *p* = 0.001).

## Discussion

In this longitudinal case-control study we found that levels of inflammatory and brain proteins did not follow different courses in patients immediately prior to or following the occurrence of delirium compared to levels in patients that did not develop delirium. However, levels of adiponectin over time remained significantly higher in patients with delirium compared to those without. In the cross-sectional analysis, we found that in patients with hypoactive delirium the levels of Tau and the ratio of Tau/Aβ_1–42_ were significantly higher compared with patients without delirium. Levels of neopterin and IL-10 were significantly higher compared to levels in patients with mixed-type delirium.

Several studies in non-ICU patients identified levels of IL-6 and IL-8 to be most strongly correlated with the development of delirium [22, 23]. Because delirium is also associated with neuronal damage, markers of neuronal damage were also found to be elevated in patients with delirium [8, 13]. Since the exact time point at which delirium occurs and its underlying pathophysiological substrate are often unclear, different markers may be elevated at different time points, based on the timing of the primary insult and the kinetics of the specific biomarker involved. This may explain why no single biomarker has consistently demonstrated an association with delirium across different patients groups and diseases with different etiology. Also, theoretically, the accuracy of biomarkers to predict the development of delirium might be better just before delirium occurs. In our study we explored the time course of several biomarkers on the days prior to and following the occurrence of delirium. While associations between many markers and delirium have been investigated, including insulin-like growth factor, S-100B, and genetic markers such as the ApoE gene [24], we advanced on our earlier data [9] and selected a relevant subset of pro-inflammatory and anti-inflammatory markers, brain-specific proteins and new promising markers in line with previous ICU studies. Based on the neuro-inflammatory hypothesis [25], we presumed that, especially before the occurrence, levels of pro-inflammatory biomarkers might show higher values in patient that develop delirium, which would strengthen the direct association between (neuro)inflammation and delirium. In contrast, no differences were observed immediately before or after delirium occurrence, suggesting that higher circulating levels of inflammatory and brain-specific markers in critically ill patients are merely markers of systemic disease severity, which in itself may be associated with delirium. This limits the potential, independent role of these biomarkers in identifying those at high risk for developing delirium as well as the early detection of delirium.

These findings are in contrast with previous biomarker studies in delirious ICU patients, with a time-series design. Daily levels of IL-6 were determined in 77 critically ill patients and higher levels of IL-6 were identified in patients with acute brain failure [15]. Lack of information on the exact time point at which delirium occurred and the inclusion of comatose patients makes a direct comparison with our study difficult. S-100B was measured on day 1 and 8 in another 63 critically ill delirious patients [16]. Higher levels of S-100B were associated with a longer duration of delirium, but no comparison with non-delirious patients was made.

We found that levels of adiponectin over time remained significantly higher in patients with delirium patients compared to patients without delirium, which is in concordance with a previous study in ICU patients [10]. Adiponectin is a protein excreted exclusively by adipocytes and it has insulin-sensitizing, vascular-protective and anti-inflammatory properties. Due to its effects on downregulation of the immune response and low adiponectin levels in patients with sepsis, some advocate therapeutic use of adiponectin in critically ill patients [26]. Although its effect on the brain is still unclear, our results intriguingly show that there is a positive association between levels of adiponectin and delirium. We did not confirm associations between levels of pro-inflammatory biomarkers over time, especially IL-6 and IL8, and delirium, even though delirious patients appeared to have more sepsis (46% vs. 20%, *p* = 0.08), a condition that has been associated with delirium [27]. Contradictory findings in ICU and non-ICU patients in relation to inflammatory markers and the development of delirium may, in addition to the timing of the sampling and the etiology of the delirium, be due to the fact that the effects of a systemic inflammatory response on the brain are also mediated by the amount of neurodegeneration [25, 28] and large differences between patient groups are likely present. In our study five patients had a history of cognitive disorders (four (11%) in the delirium group and one (7%) in the non-delirium group (*p* = 0.52)). These groups are too small to come to a conclusion based on these numbers.

We found no differences between patients with and without delirium in the levels of brain-specific proteins; however, when evaluating the clinical subtypes of delirium we found that levels of Tau protein and the ratio of Tau/Aβ_1–42_ were significantly higher in the hypoactive delirium group compared to the non-delirium group. A previous study showed that several isoforms of Aβ are associated with delirium in ICU patients without inflammation, whereby the difference in levels of Tau and the Tau/Aβ_1–42_ ratio between these groups approached statistical significance [9]. Interestingly, we found a somewhat similar association in hypoactive delirium; however, in the current study we did not differentiate between patients with and without inflammation. In recent years, the impact of subtype of delirium on various measures has been the subject of investigation. While the hypoactive form appears to have a worse outcome in terms of short-term and long-term mortality in ICU patients [3, 29], much is still unclear, especially about its association with the development of long-term cognitive disturbances.

We found levels of neopterin and IL-10 before the onset of delirium to be significantly higher in patients with hypoactive delirium compared to patients with mixed-type delirium. Neopterin, a chemotactic marker of inflammation produced by monocytes and macrophages, was found to be elevated in cerebrospinal fluid (CSF) and serum from patients with delirium [11, 30]; however, its role in critically ill patients has not previously been determined. The association between IL-10 and ICU-acquired delirium has shown conflicting results [9, 10], but up to now no discrimination between subtypes of delirium has been made. Higher values in hypoactive delirium would therefore suggest another pathophysiological mechanism compared to mixed-type delirium, but this is not supported by the absence of a difference in other inflammatory markers or by a difference in baseline characteristics in the groups. Further studies are necessary to clarify this potential association.

There are several limitations that need to be addressed. First, we measured inflammatory and brain-specific markers in peripheral blood and not in CSF. Levels of these markers and their course over time in CSF may therefore differ from those in serum and there may even be an association between markers measured in CSF and the occurrence of delirium. Second, we judged patients with one positive CAM-ICU score in the setting of sedative use as having rapid reversible sedation-related delirium [20] and therefore these patients were considered as not having delirium. While this judgment is still under debate, this only concerned one patient and considering this patient as having delirium would not have altered the outcomes. Third, we included a relatively small number of patients in a mixed medical surgical ICU, thereby possibly limiting generalization of the findings. Additionally, we did not correct for multiple testing in our statistical analysis, increasing the risk of false-positive findings. Fourth, we used the median day of delirium onset (day 3) in the non-delirium group for comparison to the patients with delirium. Since it is unknown at what time point the brain of the non-delirious would be most susceptible to delirium development, this time point was chosen in concordance with previous research [9]. Fifth, we used the CAM-ICU to detect delirium. While the specificity of the CAM-ICU is high, the sensitivity in daily practice can be rather low [31]. Nevertheless, we used the CAM-ICU three times per day, which reduces the chance of a false-negative finding in delirious patients. Finally, we did not take into account the long-term outcomes of our patients, so we cannot definitively confirm our hypothesis of an association between levels of biomarkers and long-term cognitive impairment.

## Conclusions

In this study in critically ill patients, patterns of the levels of markers of inflammation or brain proteins did not differ in delirious patients immediately prior to or following the occurrence of delirium, compared to non-delirious patients. These findings indicate that these biomarkers are of limited value in the prediction of delirium.

Levels of adiponectin over time remained significantly higher in patients with delirium compared to those without. Cross-sectional analysis of laboratory values obtained just prior to the clinical occurrence of delirium showed that serum levels of Tau protein and the ratio of Tau/Aβ_1–42_ were significantly higher in patients with the hypoactive form of delirium compared to patients without delirium. Levels of neopterin and IL-10 were significantly higher in hypoactive delirium compared to mixed-type delirium. These findings suggest different pathways in the development of these subtypes of delirium and a possible association between the hypoactive form of delirium and the development of cognitive disorders. Further studies are warranted to explore this relationship.

## Additional files

Additional file 1: Table provides an exact description of the time points of the blood sampling relative to the day of delirium occurrence and the calculation of the biomarker level one day before the onset of delirium. “t” denotes day of delirium occurrence. For example, if delirium occurred on day 2 (t), then blood sample 1 was drawn on day 1 (t-1), blood sample 2 on day 2 (t), blood sample 3 on day 4 (t + 2) and blood sample 4 on day 6 (t + 4). (PDF 72 kb)

Additional file 2: Levels of the biomarkers over time in delirious and non-delirious patients, whereby values are expressed as medians. (PDF 185 kb)

## Abbreviations

Aβ_1–40_: Amyloid β_1–40_
Aβ_1–42_: Amyloid β_1–42_
CAM-ICU: Confusion Assessment Method for the ICU CSF Cerebrospinal fluid
DLA: Dynamic light application
EDTA: Ethylenediaminetetraacetic
ELISA: Enzyme-linked immunosorbent assay
IL: Interleukin
IQR: Interquartile range
MCP: Monocyte chemoattractant protein
METOPP: Medical-ethical review on patients and trial subjects
RASS: Richmond Agitation and Sedation Scale
RM-ANOVA: Repeated-measures analysis of variance
SPSS: Statistical Package for Social Sciences
TNF: Tumor necrosis factor
