# Supplementary material for: Temporal biomarker profiles and their association with ICU acquired delirium: a cohort study
Source: Crit Care. 2018 May 25;22:137. doi: 10.1186/s13054-018-2054-5 (PMC5970442; doi:10.1186/s13054-018-2054-5)
Supplement: Supplementary file 1 — Table provides an exact description of the time points of the blood sampling relative to the day of delirium occurrence and the calculation of the biomarker level one day before the onset of delirium. “t” denotes day of delirium occurrence. For example, if delirium occurred on day 2 (t), then blood sample 1 was drawn on day 1 (t-1), blood sample 2 on day 2 (t), blood sample 3 on day 4 (t + 2) and blood sample 4 on day 6 (t + 4). (PDF 72 kb) [file 13054_2018_2054_MOESM1_ESM.pdf]

Day of delirium occurrence	Number of patients	Blood sample no.	Time points of lab samples used for longitudinal analysis	Day before delirium occurrence	Sample used for cross-sectional analysis
1	1	1	t, t+1, t+3, t+5	0	1
2	8	2	t-1, t, t+2, t+4	1	1
3	9		t-2, t-1, t+1, t+3	2	2
4	9	3	t-3, t-1, t, t+2	3	Average of samples 2 and 3
5	5		t-4, t-3, t-1, t+1	4	3
6	3	4	t-5, t-4, t-2, t	5	Average of samples 3 and 4

Additional file 1: Exact description of the time points of the blood sampling relative to the day of delirium occurrence as well as the calculation of the biomarker level one day before the onset of delirium. ‘t’ denotes day of delirium occurrence. For example, if delirium occurred on day 2 (t), then blood sample 1 was drawn on day 1 (t-1), blood sample 2 on day 2 (t), blood sample 3 on day 4(t+2) and blood sample 4 on day 6 (t+4)
