# Supplementary material for: Temporal biomarker profiles and their association with ICU acquired delirium: a cohort study
Source: Crit Care. 2018 May 25;22:137. doi: 10.1186/s13054-018-2054-5 (PMC5970442; doi:10.1186/s13054-018-2054-5)
Supplement: Supplementary file 2 — Levels of the biomarkers over time in delirious and non-delirious patients, whereby values are expressed as medians. (PDF 185 kb) [file 13054_2018_2054_MOESM2_ESM.pdf]

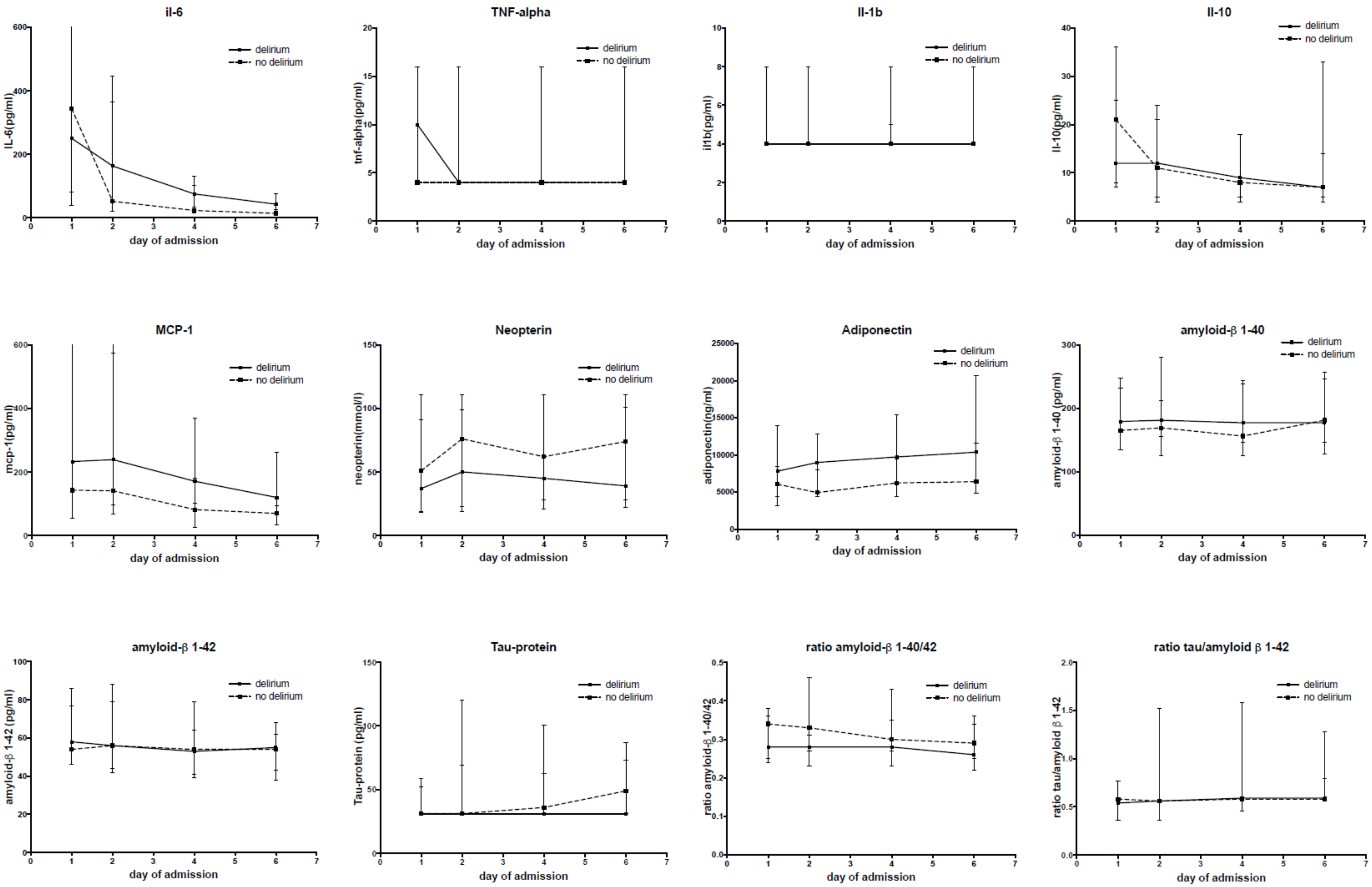

Additional file 2: levels of several biomarkers over time in delirium and non-delirium patients; values are expressed as medians
